# Differences between U23 and older semi-professional soccer players in perfectionism and passion: exploring determinant relationship between variables

**DOI:** 10.3389/fpsyg.2023.1230035

**Published:** 2024-01-16

**Authors:** Adelaida Irene Ogallar-Blanco, Eduardo García-Mármol, Filipe Manuel Clemente, Georgian Badicu, Antonio Liñán-González, Francisco Tomás González-Fernández

**Affiliations:** ^1^Department of Personality, Evaluation and Psychological Treatment, Faculty of Psychology, University of Granada, Granada, Spain; ^2^Department of Physical Education and Sports, Faculty of Sport Sciences, University of Granada, Granada, Spain; ^3^Escola Superior Desporto e Lazer, Instituto Politécnico de Viana do Castelo, Viana do Castelo, Portugal; ^4^Research Center in Sports Performance, Recreation, Innovation and Technology (SPRINT), Melgaço, Portugal; ^5^Department of Biomechanics and Sport Engineering, Gdansk University of Physical Education and Sport, Gdansk, Poland; ^6^Department of Physical Education and Special Motricity, Faculty of Physical Education and Mountain Sports, Transilvania University of Brasov, Brasov, Romania; ^7^Head of Nursing Department, Faculty of Health Sciences, University of Granada, Granada, Spain

**Keywords:** adaptive/maladaptive perfectionism, harmonious passion, obsessive passion, soccer players, U23

## Abstract

**Aim:**

Abundant studies have pointed out that perfectionism and passion are interrelated and that they both can influence performance in a variety of contexts, including sports, however, research on how these variables interact and might affect performance in team sports, such as soccer, is still scarce. The aim of this study is to analyze differences in perfectionism and passion between under-23 years-old (U23) and older semi-professional soccer players, as well as to study the possible relationship between these two psychological variables.

**Methods:**

Participants (*N* = 130 healthy semi-professional Spanish soccer players) were divided into two equivalent groups according to the participants age, U23 (*N* = 65; 21.58 ± 1.91 years) and older than 24 years (*N* = 65; 30.03 ± 3.72 years) and completed self-reports on perfectionism and passion.

**Results:**

Independent samples *t*-test determined significant differences between the U23 Group and the >24 Group in perfectionism global score and concern over mistakes (maladaptive perfectionism), and in time, value, and passion. Multiple regression analyses revealed that obsessive passion predicted maladaptive subdimensions of perfectionism, while harmonious passion predicted adaptive perfectionism.

**Discussion:**

U23 soccer players show higher levels of maladaptive perfectionism, time, value, and passion in relation with soccer than older players, probably because at this stage pressure to become professionals and to develop their technical, tactical, and physical skills is higher than in later stages.

**Conclusion:**

Identifying differences between different age groups can help professionals in tailoring their interventions and strategies to address the specific needs of athletes at different stages of development more effectively, and to optimize mental focus, reduce stress, to promote a healthy mindset for optimal performance in soccer players, furthermore, the study of moderating effects of factors such as team dynamics or coaching styles on these constructs is advised.

## 1 Introduction

Semi-professional soccer teams must, at least, have a minimum of six players under-23 years-old (U23) of a total of 22 players [e.g., Real Federación Española de Fútbol (RFEF), 2023]. This regulations regarding age grant greater opportunities for U23 players to reach professional soccer, since professional clubs often focus their efforts on recruiting and developing talent at an early age (e.g., Lim et al., 2022; Rey et al., 2023). I.e., semi-professional soccer teams include younger players with higher chances of becoming professionals (i.e., U23 players), and older and more experienced players, already at their maximum level of expertise. Therefore, examining the psychological differences between U23 and older players—differences that could increase or decrease a player’s likelihood of making it to the professional ranks of soccer—becomes an interesting task that could help us to provide appropriate guidance and the development of strategies to players on their way to the elite. The differences between younger and older soccer players have been studied in various fields, traditionally mostly focusing on physical and physiological characteristics (e.g., Santos et al., 2021; Staśkiewicz et al., 2023), although attention on age differences in psychological variables and their impact on behavior or performance has increased using a holistic approach, Kavussanu et al. (2006) found that younger players showed less antisocial and more prosocial behaviors than older players. Recently, Konter et al. (2020) found that older and more experienced soccer players showed higher levels of harmonious passion, while younger and less experienced soccer players had higher levels of obsessive passion.

Perfectionism is a personality trait that involves striving for perfection and placing excessively high expectations on oneself and others (Frost et al., 1990). The model of Frost et al. (1990), perfectionism is a multidimensional construct which includes: Personal standards (regarding exceedingly high standards of performance), concern over mistakes (the perfectionists’ concern about making mistakes and the presumably negative consequences that those mistakes would have for their self-evaluation), doubts about actions (the tendency toward indecision due to the fear of not behaving properly), parental expectations and parental criticism (perfectionists’ perceptions that their parents expect them to be perfect and that they would be disapproving if these expectations were not met, respectively) and organization (the tendency to be extremely organized and the value given to tidiness and order). The work of Hamachek (1978), differentiating between neurotic and normal forms of perfectionism, gave rise to the premise that perfectionism is a multidimensional construct with both maladaptive and adaptive components. In this regard (Frost et al., 1993), considered that concern over mistakes, parental expectations, parental criticism, and doubts about actions dimensions of perfectionism reflected the components of neurotic or maladaptive/negative perfectionism, while the dimensions of personal standards and organization would reflect normal or adaptive/positive perfectionism. Maladaptive perfectionists are individuals with extremely low tolerance for error that set unreasonably high standards. On the other hand, adaptive perfectionists are individuals that, although strive to reach moderate-to-high levels of perfectionism and set high standards, still have the capacity to feel successful and content when those standards are met, as well as to accommodate their pursuit of success and to allow minor errors in their work (Lo and Abbott, 2013; Fung et al., 2023). Perfectionism has been extensively studied in several fields, including sports, and in relation to various positive and negative psychological outcomes. For instance, studies have found that adaptive perfectionism is negatively related with negative outcomes, such as anxiety, negative affect, intentions to dropout or burnout, and positively related to sport performance, motivation, enjoyment, or team cohesion while negative dimensions of perfectionism show the opposite pattern (e.g., Mallinson-Howard et al., 2019; Deck et al., 2020; Freire et al., 2022; Olsson et al., 2022).

Perfectionism, as a tendency to set high standards for oneself, might associate to certain emotional or motivational variables, such as the experience of passion in sports. Passion is defined as a “strong inclination toward a self-defining activity that one likes (or even love), finds important (or highly value), and in which one invests time and energy” (Vallerand, 2010, p. 102). In this regard, the Dualistic Model of Passion (DMP) (Vallerand, 2010) proposes that people engage in activities to satisfy psychological needs, as competence, relatedness, or autonomy. Those activities which people find more enjoyable tend to be the preferred ones, as they fulfill these psychological needs. Few especially important activities will become passionate ones. According to the DMP, there are two types of passion: obsessive passion and harmonious passion. Obsessive passion implies engaging rigidly, excessively, and uncontrollably in the activity, usually at the expense of other life domains, due to the need to obtain certain contingences linked with it, while harmonious passion supposes an autonomous, flexible, and balanced engagement in the activity, which remains under person’s control (Schellenberg et al., 2021). In the sports context, obsessive passion has been associated with maladaptive outcomes, such as stress, injury story and injury likelihood, worry, moral disengagement, or concentration disruption, while harmonious passion relates with adaptive outcomes, as self-esteem, enjoyment, psychological well-being, flow, mastery-improvement and performance goals, cohesion, competence, autonomy or sportspersonship (e.g., Schiphof-Godart and Hettinga, 2017; Mousavi et al., 2021; Schellenberg et al., 2021; Rodrigues et al., 2022).

Being that positive subdimensions of perfectionism and passion relate to adaptive outcomes, while negative subdimensions of passion and perfectionism are usually linked to negative outcomes, the interrelation of these constructs has been recently studied in different fields. Some research has shown that the negative dimensions of perfectionism and obsessive passion and the positive dimensions of perfectionism and harmonious passion tend to relate themselves both directly (e.g., de la Vega et al., 2022) and indirectly (e.g., Emara, 2023).

Regarding sports and exercise, research of this relationship is scarcer, although a few studies have supported that perfectionism is associated to both obsessive and harmonious passion in athletes. For instance, Curran and Hill (2014) found that maladaptive dimensions of perfectionism predicted obsessive passion in a sample of young competing athletes and concluded that differences in perfectionism (maladaptive vs. adaptive) extended to the kind of passion athletes foster. In this line, Demirci and Çepikkurt (2018) also found a positive association between obsessive passion and concern over mistakes (subdimension of maladaptive perfectionism) and between harmonious passion and personal standards (subdimension of adaptive perfectionism) when studying the predictive power of these two variables on burnout in athletes. On the other hand, Schipfer and Stoll (2015) proposed in their study that perfectionism and passion might regulate exercise addiction. Although it has been found that perfectionism and passion are interrelated and that they both can influence performance in a variety of contexts, including sports, research on how these variables interact and might affect performance in team sports, such as soccer, is still scarce. This knowledge might be relevant to design and implement strategies aimed to improve performance, especially among those semi-professional players who are in a critical period of their careers, when the impact of these factors might influence their chances of reaching the professional level. Therefore, the aim of this study is to analyze the differences in perfectionism and passion between semi-professional U23 and older soccer players, as well as the interrelation shown of these two variables, to contribute to the cumulative knowledge on the matter.

## 2 Materials and methods

### 2.1 Participants

A total of 130 healthy semi-professional soccer players (25.78 ± 5.16 years, body mass: 73.75 ± 6.03 kg., height: 178.86 ± 5.81 cm.) from the Andalucía, in the south of Spain, which has a population ranging from 8,472,407 inhabitants according to the Spanish Government National Institute of Statistics (Instituto Nacional de Estadística [INE], 2020) participated in this study. This group was divided into two equivalent groups according to the participants age, one comprising those players older than 24 years-old (30.03 ± 3.72 years, body mass: 73.95 ± 5.78 kg., height: 178.54 ± 5.39 cm.) and another with players being 24 years-old or younger (21.58 ± 1.91 years, body mass, 73.55 ± 5.76 kg., height: 179.23 ± 5.71 cm.). The rationale for using 24-year-old as the division criterion was due the fact that almost all participants who were U23 players at the beginning of the season had reached 24 years-old during it. *A priori* sample size calculation was performed using a free on-line tool, G*Power^1^, with a power level of 95% and an *α* level of 0.05 and revealed that the sample size of >120 would be sufficient for the analysis.

Inclusion criteria for participant groups in this study were: (i) a background of ≥10 years of systematic soccer training and competitive experience (i.e., excluding amateur soccer players, given that reaching professional status requires long-term commitment), (ii) reporting no partial/chronic injury and no history of neuropsychological impairment that could affect the response of questionnaires, (iii) absence of potential medical problems (i.e., any type of problem that could hazard their chances of becoming professionals or could bias their psychological results), (iv) soccer players enrolled in third division RFEF in 2021–2022 season (i.e., having reached semi-professional status), (v) providing signed informed consent and (vi) complete all questionnaires.

A subsample derived from this main sample, comprising only U23 players, has been used in subsequent studies belonging to the same project derived from these findings (Martín-Moya et al., 2023).

### 2.2 Design and procedures

The present study was descriptive with a cross-sectional, correlational design with a quasi-experimental approach, and was conducted between February and March of 2023. Semi-professional soccer players usually trained a minimum of four times a week in different teams of same category (90 min ≈ per session) and played one match a week. The training sessions comprised a warm-up, main part, and cooldown.

All the soccer players were informed about the main aims of the study and completed and signed informed consent forms prior to completing the questionnaires. Athletes were treated according to the American Psychological Association guidelines for Psychological Assessment and Evaluation (American Psychological Association [APA], 2020), which ensured the anonymity of participants’ responses. In addition, the study was conducted in accordance with the ethical principles of the Helsinki Declaration for Human Research and was approved by the Ethics Committee of the School of Medicine of the University of Granada (code: 2021/64).

### 2.3 Measures

The following measures were used:

(A) Perfectionism: The Spanish version of the Frost Multidimensional Perfectionism Scale (FMPS) (Frost et al., 1990; Spanish version by Carrasco-Tornero et al., 2009), was used. This is a 35-item multidimensional measure that assesses the six specific domains of perfectionism proposed by Frost et al. (1990), with 9 items measuring the dimension concern over mistakes (e.g., *“I should be upset if I make a mistake”*), 7 items measuring personal standards (e.g., *“It is important to me that I be thoroughly competent in what I do”*), 5 items to assess parental expectations (e.g., *“My parents set very high standards for me”*), 4 items assessing parental criticism (e.g., *“Only outstanding performance is good enough in my family”*), 4 items for doubts about actions (e.g., *“I usually have doubts about the simple everyday things that I do”*) and 6 items assessing organization (e.g., *“I try to be an organized person”*). All items were answered on a 5-point Likert-type scale from 1 = hardly ever to 5 = always. The FMPS allows an overall perfectionism score as well as scores for the six subscales, the higher the values, the higher the perfectionism. The *alpha* values for this sample were 0.90 for the total FMPS, 0.83 for concern over mistakes, 0.78 for personal standards, 0.75 for parental expectations and parental criticism, 0.53 for doubts about actions, and 0.92 for organization, demonstrating high internal consistency for the total scale and five of the six subdimensions and a moderate internal consistency for doubts about actions. Appropriate psychometric characteristics have been reported for this measure in various samples (Gavino et al., 2019; Abramovitch et al., 2020).
(B) Passion was assessed using the Spanish version of the Passion Scale (Marsh et al., 2013; Spanish version by Chamarro et al., 2015). The scale comprises two six-item subscales that measure obsessive (e.g., *“I have difficulties controlling my urge to do my activity”*) and harmonious passion (e.g., *“This activity is in harmony with the other activities in my life”*) for an activity. It also includes five additional criterion-single items to determine whether participants’ involvement in the activity can be classified as a passion. These are: time (the amount of time spent on the activity), liking (love for the activity), value (how much the activity is valued), passion (the activity perceived as a passion), and identity (the activity perceived as a part of one’s identity), the latter of which was not included in earlier versions. Each question is scored on a 5-point Likert scale (1 = completely disagree, 5 = completely agree). Higher values reflect higher passion levels. The *alpha* values for this sample were 0.79 for obsessive passion and 0.84 for harmonious passion, which reflect high internal consistency for both subscales. Appropriate psychometric characteristics have been reported for this scale (e.g., Chamarro et al., 2015; Méndez-Giménez et al., 2016).

### 2.4 Procedure

The assessment protocol was available online (Google Forms®, Google LTD., USA). Participants were asked to complete the measure using their mobile phones. They received information about the study, their rights as participants, and topics including the confidentiality of their answers and how only scientific uses could be made of them. They had access to the survey only once they provided their informed consent. The participants received neither compensation nor feedback. Data were collected between February and March 2022.

### 2.5 Statistical analysis

Descriptive statistics were calculated for each variable. Before any parametric statistical analysis were performed, the assumption of normality and homoscedasticity were tested with the Kolmogorov–Smirnov and Levene’s tests, respectively. Independent samples *t*-test was used for determining differences as a repeated measures analysis (U23 Group and > 23 Group). Cohen’s *d* was the effect size indicator. To interpret the magnitude of the effect size, the following criteria were adopted: *d* ≤ 0.20, small; *d* ≤ 0.50, medium; and *d* ≤ 0.80, large (Ledesma et al., 2008). Pearson’s correlation coefficient *r* was used to examine the relationship between each one of the six subdimensions of perfectionism and the two components of passion. To interpret the magnitude of these correlations, the following criteria were adopted: Trivial: ≤0.10; small: 0.10–0.29; moderate: 0.30–0.49; large: 0.50–0.69; very large: 0.70–0.89; almost perfect: ≥0.90 (Schober and Schwarte, 2018). Multiple regression analysis was used to model the prediction of passion from the remaining variables. In this regression analysis, all variables were examined separately. Data were analyzed using Statistica software (version 13.3; Statsoft, Inc., USA).

## 3 Results

Descriptive statistics were calculated for each variable (Table 1).

**Table 1:** Descriptive statistics for the total group, *t*-test results, and effect sizes (Cohen’s *d*) comparing U23 with older soccer players.

Variable (possible scores range)	Mean ± SD [min–max]	Mean ± SD [min–max]	Mean ± SD [min–max]	*t*-test ∣ *p* value ∣Cohen’s *d*
*Frost multidimensional perfectionism scale*				
Perfectionism (35–135)	92.44 ± 15.51 [50–135]	89.63 ± 13.72 [50–119]	95.22 ± 16.73 [63–135]	*t* = −2,21, *p* = 0.03*, *d* = −0.36
Concern over mistakes (9–45)	19.08 ± 6.01 [9–40]	17.91 ± 5.26 [9–36]	20.25 ± 6.51 [10–40]	*t* = −2,24, *p* = 0.03*, *d* = −0.38
Personal standards (7–35)	23.67 ± 4.83 [10–35]	22.72 ± 4.72 [10–30]	24.62 ± 4.80 [16–35]	*t* = −1,02, *p* = 0.24, *d* = −0.40
Parental expectations (5–25)	10.41 ± 3.87 [5–23]	10.34 ± 3.95 [5–23]	10.48 ± 3.82 [5–20]	*t* = −0,19, *p* = 0.84, *d* = −2.29
Parental criticism (4–20)	5.94 ± 2.41 [4–16]	5.88 ± 2.24 [4–12]	6.02 ± 2.59 [4–16]	*t* = −0,32, *p* = 0.74, *d* = −0.05
Doubts about actions (4–20)	9.60 ± 2.50 [4–20]	9.56 ± 2.58 [4–20]	9.65 ± 2.45 [6–17]	*t* = −0,18, *p* = 0.85, *d* = −0.03
Organization (6–30)	23.72 ± 4.41 [11–30]	23.22 ± 4.59 [30–12]	24.22 ± 4.21 [30–11]	*t* = −1.28, *p* = 0.20, *d* = −0.22
*Passion scale*				
Harmonious passion (6–30)	25.37 ± 3.81 [12–30]	25.17 ± 3.66 [12–30]	25.88 ± 3.05 [18–30]	*t* = −1,18, *p* = 0.24, *d* = −0.21
Obsessive passion (6–30)	17.05 ± 5.11 [6–28]	16.34 ± 5.10 [6–28]	17.89 ± 4.98 [6–28]	*t* = −1,74, *p* = 0.08, *d* = −0.30
Time (1–5)	3.56 ± 1.14 [1–5]	3.19 ± 1.11 [1–5]	3.97 ± 1.02 [1–5]	*t* = −4,17, *p* = 0.001**, *d* = −0.74
Liking (1–5)	4.72 ± 0.0.75 [2–5]	4.66 ± 0.0.74 [2–5]	4.85 ± 0.57 [2–5]	*t* = −1,64, *p* = 0.10, *d* = −0.29
Value (1–5)	4.74 ± 0.0.67 [2–5]	4.69 ± 0.66 [2–5]	4.85 ± 0.44 [3–5]	*t* = −1,60, *p* = 0.11, *d* = −0.28
Passion (1–5)	4.58 ± 0.0.84 [2–5]	4.44 ± 0.91 [2–5]	4.78 ± 0.57 [2–5]	*t* = −2,60, *p* = 0.01*, *d* = −0.45
Identity (1–5)	4.58 ± 0.0.78 [2–5]	4.52 ± 0.82 [2–5]	4.71 ± 0.58 [3–5]	*t* = −1,54, *p* = 0.13, *d* = −0.2

^*^Denotes significance at *p* < 0.05. **Denotes significance at *p* < 0.01.

An independent samples *t*-test was used for determining differences as a repeated measures analysis (U23 Group and > 23 Group). On the one hand, a *t*-test with data from the FMPS revealed significant differences in perfectionism global score and concern over mistakes, t = −2.21, *p* = 0.03, *d* = 0.36, and *t* = −2.24, *p* = 0.03*, *d* = 0.38, respectively. However, the dataset did not reveal significant differences between personal standards, *t =* −1.02, *p =* 0.24, *d* = 0.40, parental expectation, *t =* −0.19, *p =* 0.84, *d* = −2.29, parental criticism, *t =* −0.32, *p =* 0.74, *d* = −0.05, doubts about actions, *t =* −0.18, *p =* 0.85, *d* = −0.03, and organization, *t* = −1.28, *p* = 0.20, *d* = −0.22, respectively. On the other hand, a *t-*test with data from the Passion Scale revealed significant differences in time, and passion, *t* = −4.17, *p* = 0.001, *d* = −0.74, and *t* = −2.60, *p* = 0.01, *d* = −0.45, respectively. Nevertheless, another *t*-test did not show significant differences between harmonious passion, *t* = −1.18, p = 0.24, *d* = −0.21, obsessive passion, *t* = −1.74, *p* = 0.08, *d* = −0.30, liking, *t* = −1.64, *p* = 0.10, *d* = −0.29, value, *t* = −1.60, *p* = 0.11, *d* = −0.28, and identity, *t* = −1.54, *p* = 0.13, *d* = −0.27, respectively (See Table 1).

**Figure 1:**
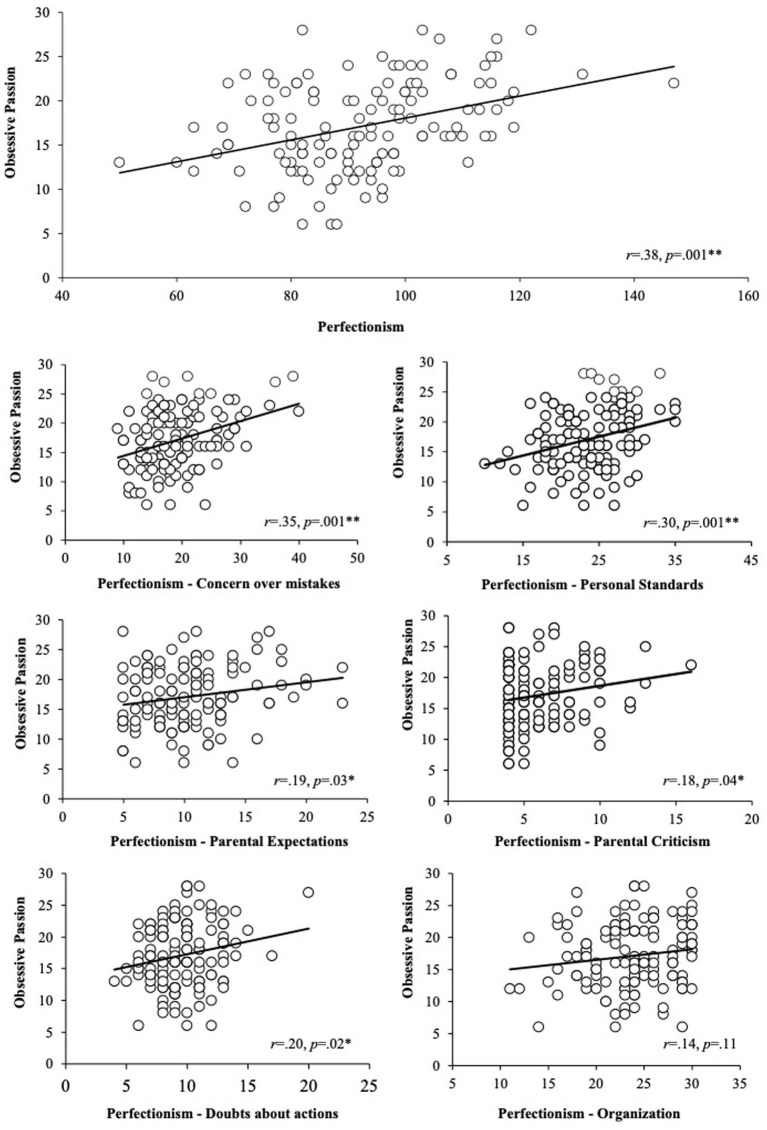
Correlation analysis between each one of the six subdimensions of perfectionism (perfectionism, concern over mistakes, personal standards, parental expectations, parental criticism, doubts about actions and organization) and obsessive passion.

Posteriorly, a correlation analysis was performed to examine the relationship between each one of the six subdimensions of perfectionism and the two components of passion. Thus, regarding passion subdimensions, several moderate correlations were found between obsessive passion and perfectionism global score, concern over mistakes, personal standards, *r* = 0.38, *p* = 0.001; *r* = 0.35, *p* = 0.001; and *r* = 0.30, *p* = 0.001, respectively. In addition, positive small correlations were revealed between obsessive passion and parental expectations, parental criticism and doubts about actions, *r* = 0.19, *p* = 0.03; *r* = 0.18, *p* = 0.04; and *r* = 0.20, *p* = 0.02, respectively. However, no significant correlations were found between obsessive passion and organization, *r* = 0.14, *p* = 0.11 (See Figure 1).

**Figure 2:**
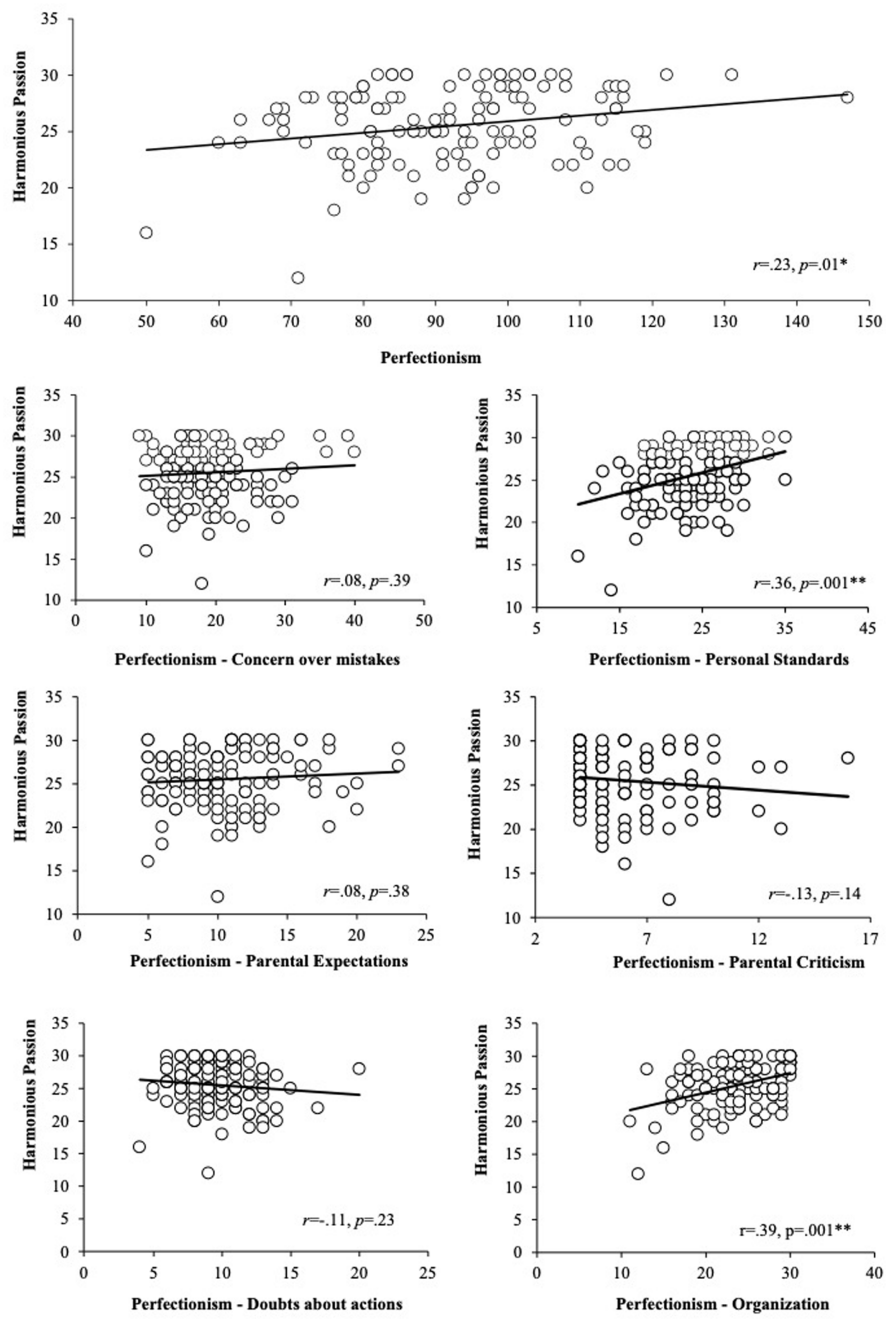
Correlation analysis between each one of the six subdimensions of perfectionism (perfectionism, concern over mistakes, personal standards, parental expectations, parental criticism, doubts about actions and organization) and harmonious passion.

Another correlations analysis revealed a positive small correlation between harmonious passion and perfectionism global score, *r* = 0.23, *p* = 0.01. Crucially, two positive moderate correlations were found between harmonious passion, and personal standards and organization, *r* = 0.36, *p* = 0.001, and *r* = 0.39, *p* = 0.001. Finally, no significant correlations were found between harmonious passion and concern over mistakes, parental expectations, parental criticism, or doubts about actions (See Figure 2).

Posteriorly, a multiple regression analysis was performed to verify which passion or perfectionism variables (in agreement with the correlation analysis), could be used to better explain the importance of different types of passion over maladaptive and adaptive dimensions of perfectionism. On the one hand, multiple regression for obsessive passion revealed significant effects for global perfectionism, concern over mistakes, personal standards, parental expectations, parental criticism, and doubts about actions (*r^2^* = 0.14, *r^2^* = 0.12, *r^2^* = 0.09, *r^2^* = 0.04, *r^2^* = 0.03, *r^2^* = 0.03, and *r^2^* = 0.04, respectively). On the other hand, multiple regression for total harmonious passion showed significant effects for global perfectionism, personal standards, and organization (*r^2^* = 0.05, *r^2^* = 0.13, and *r^2^* = 0.15, respectively).

## 4 Discussion

The aim of this study was to analyze possible differences in perfectionism and passion between U23 and older semi-professional soccer players, as well as to analyze the relationship between perfectionism and passion in these groups.

The participants were shown to be a heterogeneous group in both perfectionism and passion, with moderate-low perfectionistic characteristics, excepting for the personal standards and organization subscales, in which the opposite trend is found. Given that personal standards and organization are the perfectionism’s subdimensions considered as adaptative perfectionism (Stoeber et al., 2006, 2010; Lo and Abbott, 2013), this implies this group would be composed of moderate-adaptive perfectionists. As for passion criteria, this group shows very high levels of liking, values, passion and identity, and a moderately high value of time, which means that this group really feel passion for soccer, which is congruent, being semi-professional players. Consistent with these perfectionistic levels, the group also shows high levels of harmonious passion, while only moderate levels of obsessive passion (Curran and Hill, 2014).

Regarding differences between U23 and older players, *t*-test and Cohen’s *d*, showed some interesting results, as they revealed that U23 players showed significative differences in global perfectionism and concern over mistakes, the main subdimension of negative perfectionism. In this sense, further studies we conducted comparing levels of perfectionism among the U-23 soccer players of this sample (Martín-Moya et al., 2023) also found that youngest players showed a higher level of negative perfectionism than their older, yet U-23 companions, suggesting that perfectionism is higher among the youngest. As aforementioned, concern over mistakes constitutes the apprehension about making mistakes and about the possible negative consequences these faults could entail. This association appears logical, since U23 players are at a more critical stage of development in their personal and sporting lives than older players, and the prospect of a professional career might add an additional level of demand, since younger players still have considerable chances to become professional, chances that narrow and decrease with age. On the other hand, older players might have a different perspective, as meeting their personal levels of expectations, enjoying the sport, or maintaining their actual level, instead of focusing so much on becoming professionals. This is coherent with the differences found in time, value, and passion: U23, players are usually at the stage where the competition is most intense, so they might be fully immersed in their training, spending a significant amount of time enhancing their technical, tactical, and physical skills, while competing with other players who also are trying to become professionals. This could easily create a mindset of constant improvement and a sense of urgency to excel at every opportunity, coherent with these perfectionism and passion scores.

Correlation analyses showed moderate positive associations between obsessive passion, and concern over mistakes and personal standards, while the relation with parental expectations, parental criticism and doubts about actions were lower, and not significative in the case of organization. On the other hand, positive associations for harmonious passion and personal standards and organization were found, while the rest of perfectionism subscales did not relate with harmonious passion. As aforementioned, concern over mistakes, parental expectations, parental criticism, and doubts about actions dimensions of perfectionism are considered to reflect maladaptive/negative perfectionism, while personal standards and organization reflect adaptive/positive perfectionism (Frost et al., 1993). Maladaptive perfectionism implies the setting of unrealistic standards and a minimal tolerance for failures, whereas adaptive perfectionism allows to establish high standards but also the capacity to be flexible in the pursuit of success, disregarding small imperfections, while still and being able to feel fulfilled and satisfied when the standards are met (Lo and Abbott, 2013; Konter et al., 2020). Obsessive passion is the consequence of the internalization of the activity into one’s identity, creating the urge to engage in it over other activities or interests. On the contrary, harmonious passion is the outcome of internalizing an activity as just a component of one’s identity, which happens when one voluntarily chooses to partake in the cherished activity, free from pressure or constraints (Chamarro et al., 2015). According to these theoretical approaches, the positive relation found between maladaptive dimensions of perfectionism with obsessive passion and of adaptive perfectionism dimensions with harmonious passion, was expected and congruent with previous investigations (e.g., Curran and Hill, 2014; Demirci and Çepikkurt, 2018).

Given that the expected relationships between our variables were found, multiple regression analyses were performed to better understand these relations and the potential predictive capacity of passion on maladaptive or adaptive perfectionism. It was decided to precisely analyze the predictive power of passion on perfectionism, since it was considered easier to detect, infer, and assess passion than perfectionism by observing behaviors, especially during athletic performance. If passion can finally be shown to predict the type of perfectionism, this information could be useful when designing interventions to improve performance.

As expected, obsessive passion predicted concern over mistakes, parental expectations, parental criticism, and doubts about actions, and harmonious passion predicted personal standards and organization. For instance, obsessive passion results in over-identification with the activity in such a way that it occupies the person’s thoughts, and takes priority over other goals, even conflicting with other vital interests (Chamarro et al., 2015) while concern over mistakes reflects perfectionists’ excessive preoccupation about making mistakes and the possible negative consequences they have for their self-evaluation, and is a fundamental component of maladaptive perfectionism, having been related with lower self-esteem, negative affect, anxiety or higher inferiority feelings (Stoeber and Hotham, 2016). Therefore, this predictive capability of obsessive passion on concern over mistakes seems congruent: an individual with higher obsessive passion, would feel a stronger urge to engage in an activity (e.g., training), and a stronger need to achieve success (e.g., wining competitions). Complementary, this over identification with their sport, soccer in this case, might relate with the increase in the relevance conceded to their own mistakes, making them feel a greater threat to their sense of self, which is consistent with also displaying a higher and increased perception of potential errors and an inclination to focus on mistakes rather than successes, which is paradigmatic of concern over mistakes. The relation with the rest of negative perfectionism dimensions (especially doubts about actions) is also consistent with this same approach, the smaller predictive capability might be because this sample of semi-professional soccer players shows moderate perfectionism, being plausible that the effect is more salient in the concern over mistakes dimension, core in maladaptive perfectionism, than in the other dimensions, especially parental expectations and parental criticism, related with parental (thus external) demands.

Moreover, predictive power of harmonious passion on positive dimensions of perfectionism (personal standards and organization) is coherent, since both compliment themselves: Harmonious passion is motivated by love and enjoyment of an activity, rather than by rewards or pressures from the outside (Chamarro et al., 2015) and has also been related to well-being enhancement, positive affect, and less anxiety and depression (Carpentier et al., 2012). In addition, personal standards relate with the set of principles and beliefs, such as responsibility or integrity, that someone uses to direct their actions and choices, which makes these individuals more able to find a gist of meaning and satisfaction in their lives, by upholding personal principles, while organization refers to the emphasis in orderly and neatness (Carrasco-Tornero et al., 2009; Stoeber and Hotham, 2016). Positive perfectionism has been related to positive outcomes, such as higher levels of self-efficacy, positive wellbeing, self-esteem, greater life satisfaction, and higher internal locus of control (Lo and Abbott, 2013). Players who actively and harmoniously pursue their passions, making sure that their actions are consistent with their values and beliefs, and that feel a stronger sense of fulfillment and joy by passionately engaging in their sport would more likely feel more fulfilled and have a clearer sense of purpose in life, which is coherent with higher levels of personal standards and organization. Consequently, since both positive subdimensions of perfectionism and harmonious passion contribute to a person’s fulfillment in life, the positive relation found is logical and complementary. For this same reason, the lack of relation between obsessive passion and organization seems concordant.

Our results are useful for better understanding the relationship between obsessive and harmonious passion with maladaptive and adaptive perfectionism, which could be convenient for decision making and the prediction of future outcomes (e.g., in burnout Woods et al., 2022). For instance, knowing that obsessive passion can predict maladaptive perfectionism and harmonious passion can predict adaptative perfectionism, may have some important practical implications. Firstly, obsessive passion shows a characteristic behavioral pattern branded by a strong commitment and dedication to an activity that includes certain propension to rigidity and inflexibility, urgency, and necessity to engage in the activity and achieve success (Schellenberg et al., 2021; Rodrigues et al., 2022), especially in young players, all of them being characteristics that usually become manifest through behaviors that could be easily identifiable to a trained coach. Awareness of the importance of detecting this type of behavioral patterns in players, could help the early identification of individuals who may be at risk and facilitate taking preventive measures to avoid or hinder the reinforcement of maladaptive perfectionistic patterns. It could also help to detect those older players who already experience maladaptive perfectionism, so interventions aimed to reduce the possible negative outcomes can be implemented (e.g., fostering a healthier relationship with sport and competition during trainings). Additionally, when harmonious passion behavioral patterns are detected, coaches, parents, health psychologists and educators, conscious of the potential associations with adaptive perfectionism, might help enhancing this healthy and productive passion, promoting dedication and healthy commitment with soccer, thus facilitating the enjoyment and motivation of players.

Despite the contributions of this study, there are several limitations that should be mentioned and conveniently addressed in future research. Future studies should increase the number and heterogeneity of participants in order to enhance the generalizability of the results. This research is focused on semi-professional soccer players, and the findings obtained herein might not be applicable to other athletes. In addition, our findings can be different to those obtained in younger, mid-aged and older players of different categories or sports. Lastly, this is a descriptive study with a cross-sectional, correlational design that was limited to finding associations and predictions between passion and perfectionism subdimensions. Thus, our findings should be replicated and expanded using other research designs and analytical techniques.

## Data availability statement

The raw data supporting the conclusions of this article will be made available by the authors, without undue reservation.

## Ethics statement

The studies involving humans were approved by ethics committee (code: 2021/64). The studies were conducted in accordance with the local legislation and institutional requirements. The participants provided their written informed consent to participate in this study. Written informed consent was obtained from the individual(s) for the publication of any potentially identifiable images or data included in this article.

## Author contributions

FG-F and AO-B: conceptualization and methodology. FG-F: formal analysis and supervision. FG-F, AO-B, GB, AL-G, EG-M, and FC: writing—original draft preparation. EG-M, GB, and FC: Writing—review and editing. All authors have read and agreed to the published version of the manuscript, contributed equally to the manuscript, read and approved the final version of the manuscript.

## Footnotes

^1^ www.gpower.hhu.de
